# CD38 Deficiency Protects Mice from High Fat Diet-Induced Nonalcoholic Fatty Liver Disease through Activating NAD^+^/Sirtuins Signaling Pathways-Mediated Inhibition of Lipid Accumulation and Oxidative Stress in Hepatocytes

**DOI:** 10.7150/ijbs.65588

**Published:** 2021-10-17

**Authors:** Lin Xie, Ke Wen, Qian Li, Cong-Cong Huang, Jia-Le Zhao, Qi-Hang Zhao, Yun-Fei Xiao, Xiao-Hui Guan, Yi-Song Qian, Lu Gan, Ling-Fang Wang, Ke-Yu Deng, Hong-Bo Xin

**Affiliations:** 1 National Engineering Research Center for Bioengineering Drugs and the Technologies, Institute of Translational Medicine.; 2 School of Pharmacy, Nanchang University, Nanchang 330031, P.R. China.; 3 School of Life Science, Nanchang University, Nanchang 330031, P.R. China.; 4 Research Laboratory of Emergency Medicine, Department of Emergency Medicine, West China Hospital, Sichuan University, Chengdu 610041, P.R. China.

**Keywords:** CD38, Sirtuins, peroxisome proliferator-activated receptor α, oxidative stress, lipid accumulation

## Abstract

Nonalcoholic fatty liver disease (NAFLD) is characterized by excessive lipid accumulation in hepatocytes. CD38 was initially identified as a lymphocyte surface antigen and then has been found to exist in a variety of cell types. Our previous studies showed that CD38^-/-^ mice were resistant to high-fat diet (HFD)-induced obesity. However, the role and mechanism of CD38 in HFD-induced NAFLD is still unclear. Here, we reported that CD38^-/-^ mice significantly alleviated HFD-induced hepatic steatosis. HFD or oleic acid (OA) remarkably increased the mRNA and protein expressions of CD38 in mouse hepatic tissues and primary hepatocytes or hepatic cell lines *in vitro* and *in vivo*, suggesting that CD38 might play a role in HFD-induced hepatic steatosis. We observed that CD38 deficiency markedly decreased HFD- or OA-induced the lipid accumulation and oxidative stress in CD38^-/-^ livers or primary hepatocytes, respectively. In contrast, overexpression of CD38 in Hep1-6 cells aggravated OA-induced lipid accumulation and oxidative stress. Furthermore, CD38 deficiency markedly inhibited HFD- or OA-induced the expressions of NOX4, and increased the expression of PPARα, CPT1, ACOX1 and SOD2 in liver tissue and hepatocytes from CD38^-/-^ mice, indicating that CD38 deficiency-mediated the enhancement of fatty acid oxidation and the inhibition of oxidative stress contributed to protecting NAFLD. More importantly, Ex527 (Sirt1 inhibitor) and 3-TYP (Sirt3 inhibitor) significantly enhanced OA-induced lipid accumulation and oxidative stress in CD38^-/-^ primary hepatocytes, suggesting that the anti-lipid accumulation of CD38 deficiency might be dependent on NAD/Sirtuins-mediated enhancement of FAA β-oxidation and suppression of oxidative stress in hepatocytes. In conclusion, we demonstrated that CD38 deficiency protected mice from HFD-induced NAFLD by reducing lipid accumulation and suppressing oxidative stress via activating NAD/Sirtuins signaling pathways.

## Introduction

Nonalcoholic fatty liver disease (NAFLD) including nonalcoholic fatty liver (NALF) and nonalcoholic steatohepatitis (NASH) is a clinic syndrome characterized by excessive intrahepatic fat deposition and caused by many factors except alcohol and other well-defined liver injury factors 1. NAFL may develop NASH if there is any inflammation in liver, whereas NASH may be further developed to hepatic cirrhosis or liver cancer 2-4. It has been reported that the prevalence rates of NAFLD were from 6 to 35% in the general population from different countries, with a median of 20%, which was closely related to genetic background, economy, environment and lifestyle 5. More importantly, the high prevalence of NAFLD may be linked to the metabolic abnormities such as obesity and diabetes 6.

CD38 (Cluster of Differentiation 38) is a single-chain type II transmembrane glycoprotein with a short N-terminal cytoplasmic domain and a long C-terminal extracellular domain 7. It was first identified in lymphocytes as a lymphocyte-specific antigen and later numerous studies further found that the protein not only distributed to a variety of cell types, but also existed in the intracellular membranes such as nuclear membrane, mitochondria and endoplasmic reticulum 8. CD38, as a multifunctional enzyme, is responsible for degradation of intracellular NAD^+^ and the generation of the second messengers such as cyclic ADP-ribose in mammalian cells, in which CD38 has been implicated in the regulation of physiological functions such as maintaining intracellular NAD^+^ homeostasis and modulating intracellular Ca^2+^ signals, and in the occurrence of numerous diseases such as cardiovascular diseases, aging, obesity, diabetes and inflammation at the pathological conditions 7, 8. Sirtuins proteins are a type of NAD^+^ dependent deacetylase, which is involved in many cellular and physiological functions. Studies showed that the expressions or activities of most sirtuins in NAFLD were down-regulated 9 and CD38 was a key regulator in cardiovascular disease and metabolic syndrome through NAD^+^/Sirtuins signaling 10. Our previous studies showed that CD38^-/-^ mice were resistant to AngII-induced cardiac hypertrophy, significantly protected heart from ischemia/reperfusion injury and high fat diet-induced oxidative stress, and remarkably inhibited AngII-induced smooth muscle cell and cardiomyocyte senescence 11-15. However, whether CD38 affects NAFLD and the underlying mechanism remains unknown.

In the present study, we observed that CD38 deficiency significantly alleviated HFD- or oleic acid (OA)-induced hepatic lipid accumulation and oxidative stress *in vivo* and *in vitro*. In contrast, overexpression of CD38 aggravated OA-induced hepatic steatosis and oxidative stress. Furthermore, we demonstrated that the protective role of CD38 deficiency on NAFLD was related to the inhibition of lipid accumulation and oxidative stress through activating Sirtuins signaling pathways.

## Materials and Methods

### Animals

Male CD38^-/-^ mice at the age of 6-8 weeks in C57BL/6J background were used for this study. The age-matched WT mice with C57BL/6J background were used as the control groups. CD38^-/-^ mice were kindly provided by Dr. Frances E. Lund (Rochester). The experimental protocols were approved by the Ethics Committee of Nanchang University and the experiments involved in animals were carried out in accordance with the Guide for the Care and Use of Laboratory Animals of Nanchang University. Mice with the age of 8 weeks had free to access water and were fed with either normal diet (ND) or high fat diet (HFD) (60% HFD, D12492; Research Diets Inc. New Brunswick, NJ, USA) *ad libitum* for 12 weeks.

### Cell culture and treatment

Primary hepatocytes were isolated from male mice with age of 6-8 weeks following the perfusion protocol as previously described 16. At 70%-80% confluence, the cells were cultured with the fresh media containing 0.5 mM Oleic acid (OA). After 24 h treatment, the cells were harvested for further experiments. Hep1-6 cell were cultured in 6-well plates in Dulbecco's modified Eagle's medium (DMEM) (Gibco, Grand Island, NY, USA) with 10% fetal bovine serum (FBS, Gibco) and 1% penicillin/streptomycin (Gibco) in a 5% humidified CO2 incubator at 37 °C. The cells were used for the experiments when they were in the exponential phase of growth.

### Triglyceride measurement

The intracellular and tissue triglycerides were measured using triglyceride assay kit (PPLYGEN, Beijing, China) according to the manufacturer's protocol. Briefly, cells were seeded into 6-well plates and treated with 0.5 mM OA for 24 h, the cells were washed with PBS, and then lysed by homogenizer. Tissues were lysed by homogenizer too. The samples were stood for 10 min, and the supernatant was heated at 70 °C for 10 min and then centrifuged at 2000 rpm for 5 min. Absorbance was measured at 550 nm using a microplate reader. The results were normalized by the total protein concentrations. All experiments were performed at least three times.

### Measurements of SOD activities

SOD activities were detected using SOD Assay Kit-WST (Dojindo, mashiki-machi, Japan) according to the manufacturer's protocol. Briefly, the cells were seeded into 6-well plates and treated with 0.5 mM OA for 24 h, and then the cells were washed with PBS, and lysed by ultra-sonication. Tissues were lysed by ultra-sonication too. The samples were centrifuged at 10000 × g for 10 min at 4 °C. The supernatants were used for SOD activity (relative activity) measurement according to the instruction. Absorbance was measured at 450 nm using a microplate reader. All experiments were performed at least three times.

### Measurement of peroxidation levels

The contents of intracellular and tissues MDA were measured using MDA assay kit (Nanjing Jiancheng Bioengineering Institute, Nanjing, China) according to the manufacturer's protocol. Briefly, cells were seeded into 6-well plates and treated with 0.5 mM OA for 24 h, and then washed with PBS, and lysed by homogenizer. Tissues were lysed by homogenizer too. MDA reacts with thiobarbituric acid (TBA) at 90-100 °C with an acidic condition. The reactions yield a pink MDA-TBA conjugate, and the absorbance of the supernatants were measured at 532 nm using a microplate reader. Protein concentration in lysates was determined by BCA Protein Assay Kit (Pierce). All experiments were performed at least three times.

### Mitochondrial membrane potential assay

Mitochondrial membrane potential (MMP) was detected by MMP assay kit following the manufacturer's protocol (Beyotime Biotechnology, Shanghai, China). Briefly, the cells were seeded into 6-well plates and treated with 0.5 mM OA for 24 h, and then stained with JC-1 dye for 20 min, and washed with staining buffer. The fluorescence was detected with automatic microplate reader at wavelengths of 490 nm (excitation), 530 nm (emission) and 525 nm (excitation), 590 nm (emission) respectively. All experiments were performed at least three times.

### Haematoxylin and eosin (H&E) staining

The histological analysis was performed using H&E staining. Briefly, the liver tissues were isolated from mice after euthanasia, fixed overnight in 4% formalin then embedded in paraffin blocks. The samples were sliced into 5 μm sections and were stained with hematoxylin and eosin (H&E) according to standard protocol.

### Oil Red O staining

The lipid analysis was performed using Oil Red O staining. Briefly, the cells were washed with phosphate buffered saline (PBS) and fixed with 4% paraformaldehyde for 1 hr at room temperature. The cells then were stained with Oil Red O for 30 min using a 60:40 (v/v) dilution in water with a 0.5% stock solution (in isopropanol). Finally, the cells were examined by light microscopy. For quantitative analysis, Oil red O was eluted using 100% isopropanol, and the lipid formation was measured with absorbance at 490 nm.

### Western blotting

Liver tissues and cells were lysed with RIPA buffer (added with 1mM PMSF). Lysates were centrifuged at 13,000 rpm for 15 min at 4°C. Protein concentration in lysates was determined by BCA Protein Assay Kit. The samples were separated by 10% SDS-PAGE and then transferred to PVDF membrane. The membrane was blocked with 5% non-fat milk for 60 min and probed with indicated antibodies. The antibodies of CD38 (R&D), SOD2 (CST), Cat (CST), NOX4 (Abcam), SIRT1 (CST), PPARα (santa Cruz), ACOX1 (Abcam) and CPT1α (Abcam) were used at 1:1000 dilution, respectively. The membrane was then incubated with HRP-conjugated secondary antibody at 1:5000 dilutions for 1 hour at room temperature and visualized by ECL system (Fdbio science, Hangzhou, China).

### Quantitative real time PCR analysis (qRT-PCR)

Total RNAs were extracted from tissues and cells with Trizol (Invitrogen, Carlsbad, CA, USA) followed by DNase treatment. RNA concentration was measured by Nano 2000 (Thermo Fisher). Then RNA was reversely transcribed using the Takara high capacity cDNA synthesis kit (TaKaRa, Dalian, China) according to the manufacturer's instructions and the cDNAs were stored at -80 °C until use. Quantitative PCR was performed using the ABI-ViiA7 PCR machine. Relative expression of mRNAs was determined after normalization with GAPDH levels using the ∆Ct method. Primers used for real-time PCR are shown below: GAPDH (F-AGCCAAAAGGGTCATCATCT; R-GGGGCCATCCACAGTCTTCT), CD38 (F-CTGCCAGGATAACTACCGACCT; R-CTTTCCCGACAGTGTTGCTTCT), MCAD (F- GATCGCAATGGGTGCTTTTGATAGAA; R- AGCTGATTGGCAATGTCTCCAGCAAA), CPT1α (F- ACATCCCTAAGCAGTGCCAGTT; R- TCGTCCGGCACTTCTTGATC), PPARα (F- GGGTACCACTACGGAGTTCACG; R- CAGACAGGCACTTGTGAAAACG), Sirt1 (F- GATGACGATGACAGAACGTCACA; R- GGATCGGTGCCAATCATGAG), ACOX1 (F- GCCTGAGCTTCATGCCCTCA; R- ACCAGAGTTGGCCAGACTGC).

### Statistical analysis

The results were presented as mean ± SEM. Student's t test and one-way analysis of variance (ANOVA) were used for comparation of two or multiple groups, respectively. Statistical significances were showed as *p < 0.05, **p < 0.01 and ***p < 0.001.

## Results

### High fat diet up-regulates the expression of CD38 in mouse livers

**Figure 1:**
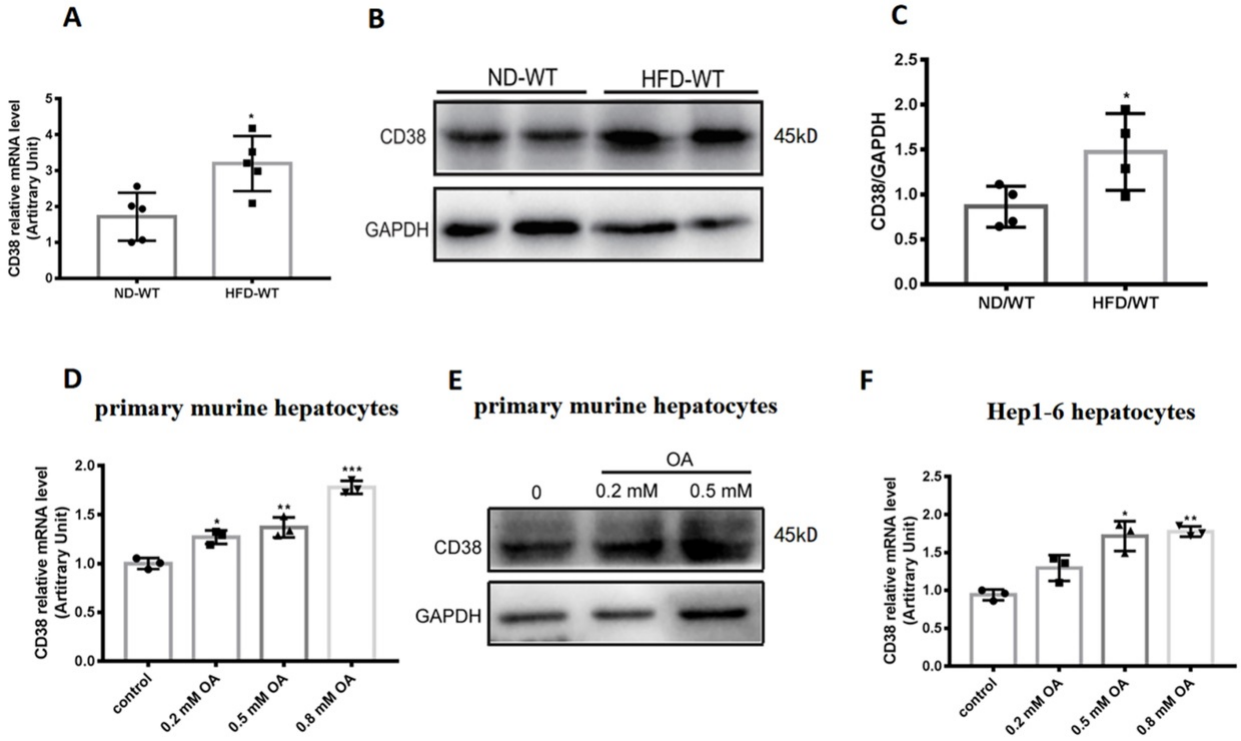
** CD38 expression was up-regulated by high fat diet or oleic acid in liver tissue or hepatocytes. (A)** CD38 mRNA expressions were determined by RT-PCR in liver tissue from WT mice fed with 12 weeks of ND or HFD. CD38 protein expressions were examined by western blot **(B)** and quantitative analysis **(C)** in liver tissue from WT mice fed with 12 weeks of ND or HFD. **(D)** CD38 mRNA expressions were determined by RT-PCR in primary hepatocytes treated with different concentrations of OA for 24 hours. **(E)** CD38 protein expressions were determined by western blot in primary hepatocytes treated with different concentrations of OA for 24 hours and** (F)** CD38 mRNA expressions were examined by RT-PCR in Hep1-6 cells treated with different concentrations of OA for 24 hours. Data are shown as means ± SEM, *p<0.05, **p<0.01 and ***p<0.001, n = 3~5 per group.

To determine the roles of CD38 in hepatic lipid metabolism, we first examined the CD38 expressions in liver tissue from the mice fed with high fat diet (HFD). The results showed that HFD significantly up-regulated the expressions of CD38 mRNA and protein in liver tissues from the mice fed with HFD compared with the mice fed with normal diet (ND) (Figure 1A-C). To further confirm the results *in vivo*, the expressions of CD38 were also examined with OA stimulation in primary hepatocytes which were isolated from adult wild type mice. The results showed that the mRNA and protein levels of CD38 were increased in a dose-dependent manner after OA stimulation (Figure 1D and 1E). Moreover, we also observed that OA triggered a marked increase in CD38 expression in mouse hepatoma Hep1-6 cells (Figure 1F). These results suggested that CD38 might play a role in the development of HFD-induced NAFLD in mice.

### CD38 deficiency alleviates HFD-induced hepatic steatosis

**Figure 2:**
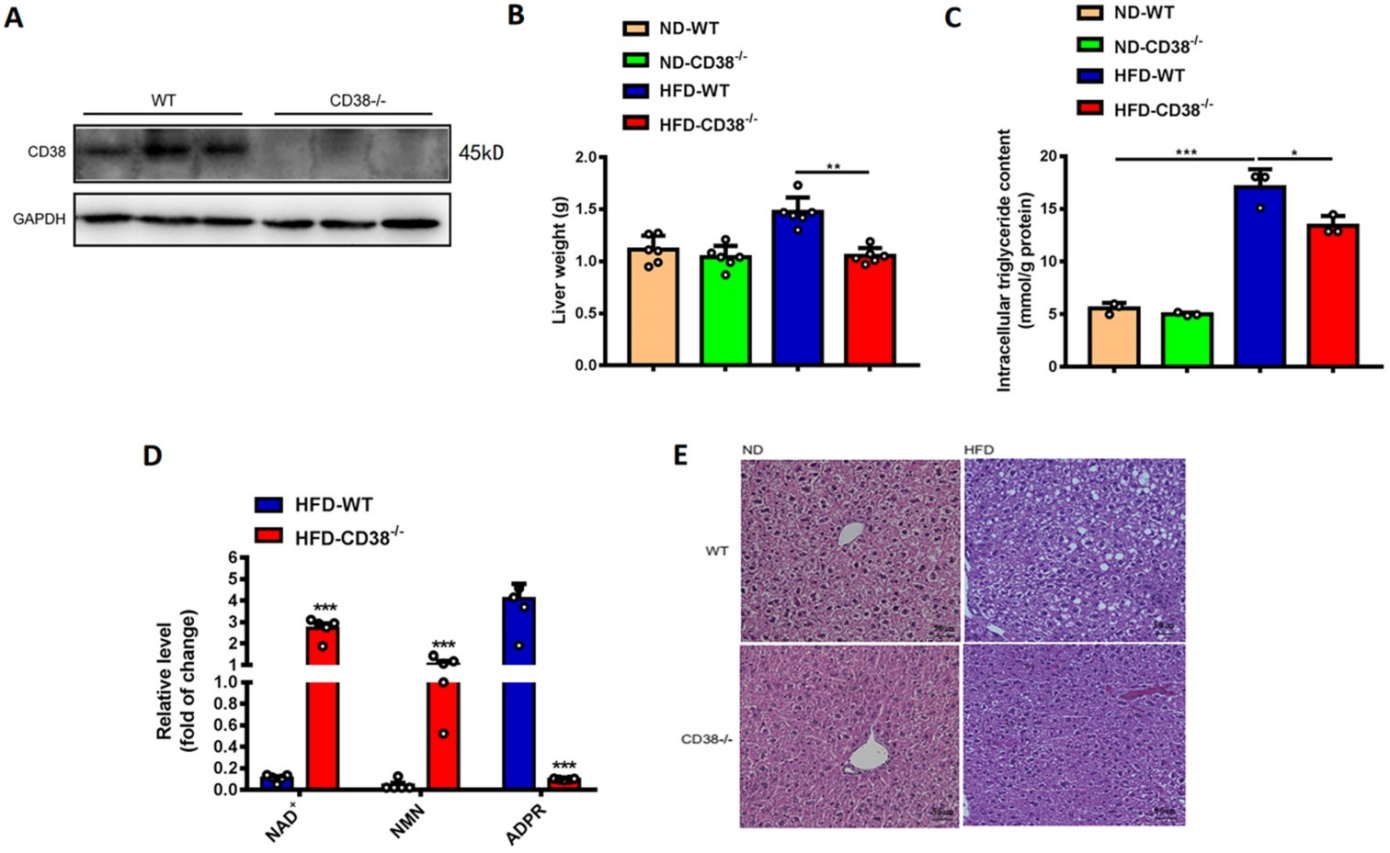
** CD38 deficiency alleviated HFD-induced hepatic steatosis in mice. (A)** The efficiency of CD38 knockout was confirmed by Western Blot. **(B)** The liver weights of WT and CD38^-/-^ mice were measured at 12 weeks post-HFD administration (n=5). **(C)** The intracellular triglyceride (TG) levels were quantitatively measured in liver tissues from CD38-/- and WT mice fed with 12 weeks of HFD. **(D)** The relative concentrations of nicotinamide adenine dinucleotide (NAD^+^), nicotinamide mononucleotide (NMN) and ADP-ribose were measured in livers from CD38^-/-^ and wild type mice treated with HFD. **(E)** The morphological alterations were analyzed by H&E staining in liver tissue from CD38^-/-^ and WT fed with 12 weeks of ND or HFD. Data are shown as means ± SEM, *p<0.05, **p<0.01 and ***p<0.001, n = 6-7 per group.

We previously observed that deletion of CD38 significantly inhibited HFD-induced obesity in mice 12. Here, we first examined the effects of CD38 deficiency on HFD-induced hepatic steatosis in CD38^-/-^ mice. As showed in Figure 2A, the deletion of CD38 was confirmed by western blot analysis. In addition, the results showed that CD38 deficiency remarkably reduced HFD-induced increases of liver weights (Figure 2B) and intracellular triglycerides in hepatic tissues (Figure 2C) compared with control group. CD38 is the main hydrolase of cellular NAD^+^, therefore, we also examined the intracellular contents of multiple metabolites including NAD^+^ in livers of CD38^-/-^ mice fed with HFD. The results showed that deletion of CD38 markedly elevated the levels of NAD^+^ and NAD^+^ precursor nicotinamide mononucleotide (NMN), whereas the metabolite ADP-ribose was significantly reduced in CD38^-/-^ livers under HFD induction (Figure 2D). Moreover, H&E staining results showed that the hepatic lipids in CD38^-/-^ mice fed with HFD were much lesser than that of WT mice fed with HFD, whereas there was no significant difference between CD38^-/-^ and WT mice fed with ND (Figure 2E). These results indicated that CD38 deficiency significantly ameliorated HFD-induced hepatic lipid accumulation *in vivo*.

### CD38 deficiency increases fatty acid oxidation and decreases oxidative stress in liver

**Figure 3:**
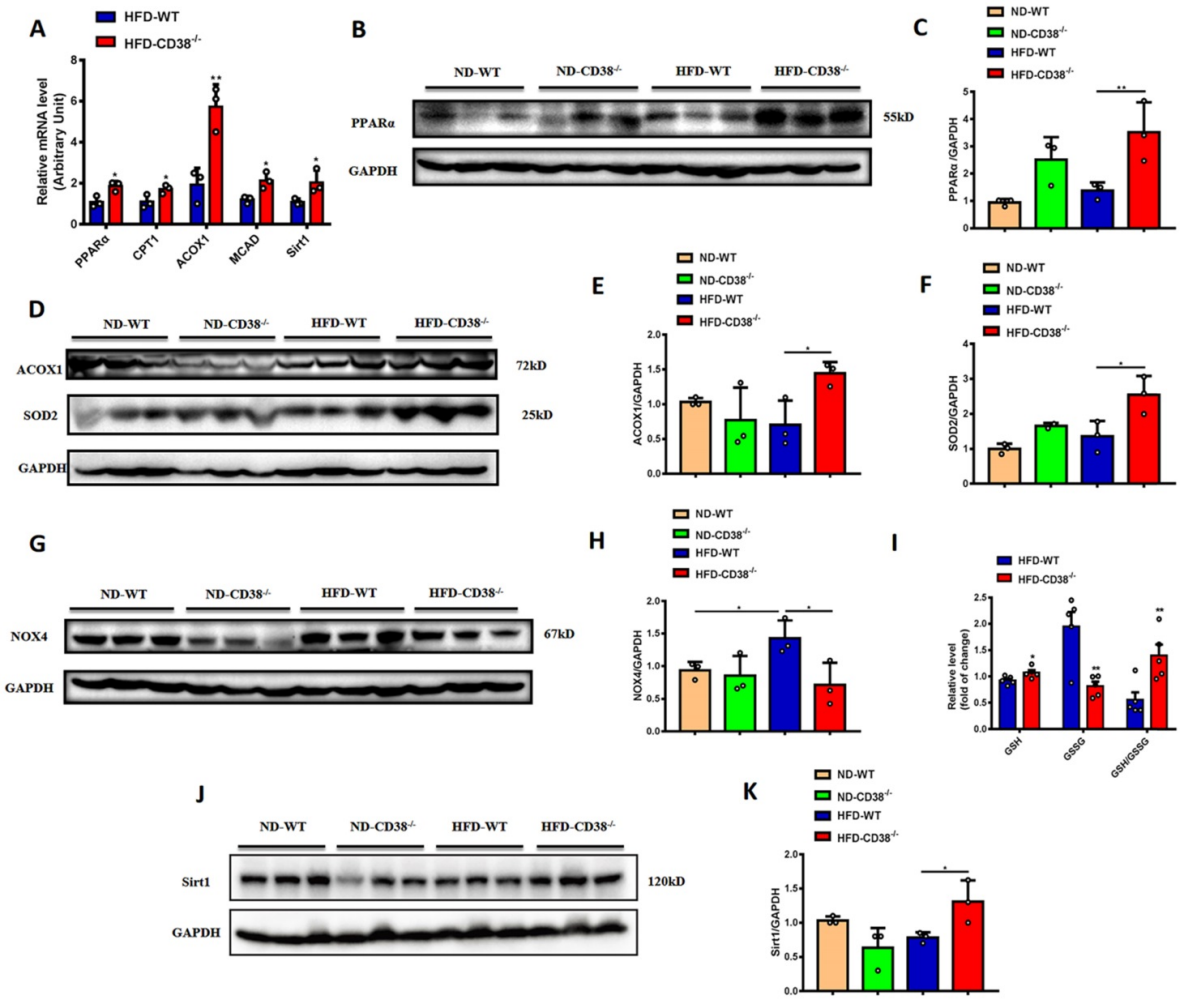
** CD38 deficiency enhanced fatty acid oxidation and inhibited oxidative stress in mouse liver tissues. (A)** The expressions of the fatty acid oxidation genes such as PPARα, CPT1, ACOX1 and MCAD, and Sirt1 were quantitatively analyzed by RT-PCR in liver tissue from CD38^-/-^ and WT mice fed with HFD. The protein expressions of PPARα **(B, C)**, ACOX1 **(D, E)** and Sirt1 **(J, K)** were confirmed by western blot analysis in liver tissue from CD38^-/-^ and WT mice fed with HFD. **(G)** The expressions of SOD2 **(D, F)** and NOX4 **(G, H)** proteins were determined by western blot analysis in liver tissue from CD38^-/-^ and WT mice fed with HFD. **(I)** The relative concentrations of GSH, GSSG and GSH/GSSG ratio were measured in liver tissue from CD38^-/-^ and WT mice fed with HFD. Data are shown as means ± SEM, *p<0.05 and **p<0.01, n = 3-5 per group.

It has been reported that the increase of fatty acid oxidation alleviated the HFD-induced NAFLD 17. In order to elucidate the mechanisms of the anti-hepatic steatosis of CD38 deficiency, we first examined the expressions of the genes involved in fatty acid oxidation in liver tissues from CD38^-/-^ mice fed with HFD. The results showed that the mRNA expressions of PPARα, CPT1, ACOX1 and MCAD were significantly increased in liver tissues in HFD-treated CD38^-/-^ mice compared with WT mice (Figure 3A), in which the protein expressions of PPARα (Figure 3B and 3C) and ACOX1 (Figure 3D and 3E) were confirmed by western blot analysis, suggesting that CD38 deficiency-mediated inhibition of lipid accumulation might be related to the increase of fatty acid oxidation.

Mounting evidences indicate that oxidative stress is closely related to the development of HFD-induced NAFLD. Next, we examined the expressions of SOD2 and NOX4, two key proteins involved in oxidative stress, in liver tissues in CD38^-/-^ and WT mice fed with HFD. The results showed that disruption of CD38 significantly increased the expression of SOD2 (Figure 3D and 3F), and decreased the expression of NOX4 (Figure 3G and 3H) in liver tissues from CD38^-/-^ mice compared with WT mice fed with HFD. In addition, CD38 deficiency significantly reversed HFD-induced the increase of the reduced glutathione (GSH) and decrease of the oxidized glutathione (GSSG) in liver tissues from CD38^-/-^ mice compared with WT mice (Figure 3I). Especially, the hepatic GSH/GSSG ratio was remarkably increased in CD38^-/-^ mice compared with WT mice, suggesting that CD38 deletion may suppress HFD-induced oxidative stress *in vivo* (Figure 3I). These results demonstrated that CD38 deficiency increased the expressions of the genes related to fatty acid oxidation and alleviated HFD-induced the increase of oxidative stress *in vivo*. Sirt1 is a NAD^+^-dependent protein deacetylase which deacetylates many target proteins such as PPARα and SOD2. It has been reported that hepatocyte-specific deletion of Sirt1 aggravated hepatic steatosis 18. In our study, the mRNA and protein expressions of Sirt1 were significantly increased in hepatic tissues of CD38^-/-^ mice compared with WT mice fed with HFD (Figure 3A, 3J and 3K), suggesting that CD38 deficiency-mediated the increase of fatty acid oxidation and inhibition of oxidative stress might be associated with activating sirtuins signaling pathways.

### CD38 deficiency decreases lipid accumulation and oxidative stress in hepatocytes *in vitro*

**Figure 4:**
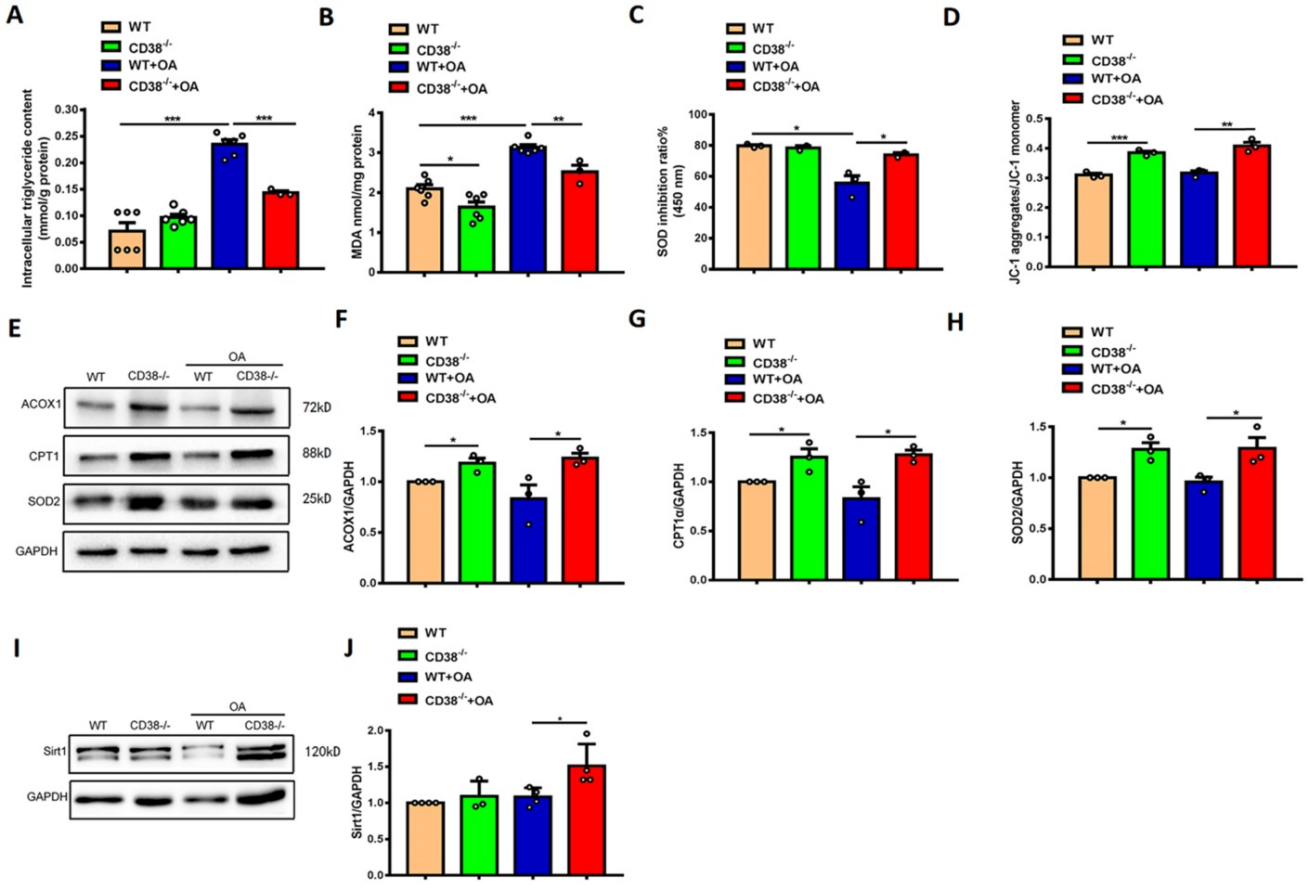
** CD38 deficiency alleviated OA-induced lipid accumulation and oxidative stress in hepatocytes.** The intracellular triglyceride **(A)**, MDA formation **(B)**, SOD activity **(C)** and mitochondrial membrane potential **(D)** were determined in primary hepatocytes from WT and CD38^-/-^ mice treated with OA or vehicle. The expressions of ACOX1** (E, F)**, CPT1 **(E, G)**, SOD2 **(E, H)** and Sirt1 **(I, J)** were examined by western blot analysis in primary hepatocytes from WT and CD38^-/-^ mice. Data are shown as means ± SEM, *p<0.05, **p<0.01 and ***p<0.001, n = 3 per group.

To further clarify the protective roles of CD38 deficiency in HFD-induced NAFLD *in vivo*, we examined the effects of CD38 deficiency on OA-induced lipid accumulation and oxidative stress in the primary hepatocytes isolated from adult mice. As showed in Figure 4A-4C, CD38 deficiency significantly reduced OA-induced the increases of the intracellular TG contents and MDA formation in hepatocytes in CD38^-/-^ mice compared with WT mice. Moreover, lack of CD38 reversed OA-induced the reduction of the SOD activity in primary hepatocytes from CD38^-/-^ mice (Figure 4C), suggesting that deletion of CD38 was able to attenuate OA-induced oxidative stress in hepatocytes. It has been reported that the mitochondrial dysfunction played an important role in NAFLD. In our results, we observed that mitochondrial membrane potential was increased in CD38 deficient hepatocytes with or without OA stimulation, suggesting that CD38 deletion could reduce lipid-induced mitochondrial damage *in vitro* (Figure 4D). Our results also showed that the expressions of the genes involved in fatty acid oxidation and oxidative stress such as ACOX1, CPT1 and SOD2 were markedly increased in CD38^-/-^ hepatocytes (Figure 4E-4H). Furthermore, OA stimulation significantly increased Sirt1 expression (Figure 4I-4J) in CD38^-/-^ hepatocytes compared with the ones from WT mice. These results suggested that CD38 deficiency-mediated inhibitions of hepatic lipid accumulation and oxidative stress may be partially related to increasing Sirt1 expression.

### Overexpression of CD38 aggravates hepatocytic lipid accumulation *in vitro*

**Figure 5:**
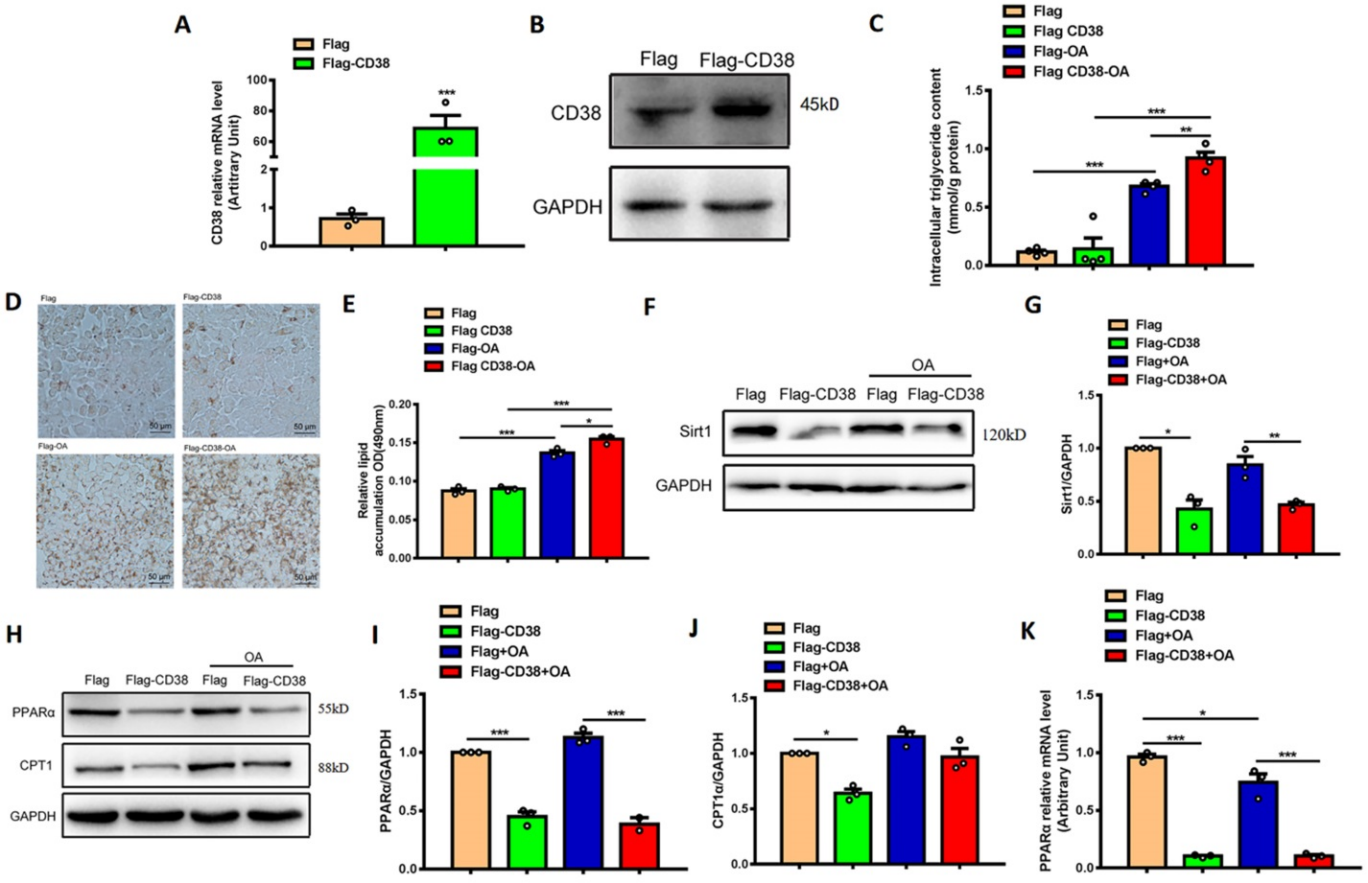
** Overexpression of CD38 aggravated OA-induced lipid accumulation in hepatocytes *in vitro*. (A)** The expressions of CD38 were determined by real-time PCR in Hep1-6 cell lines transfected with expressing vector of CD38. **(B)** The expression of CD38 protein was confirmed by western blot analysis in Hep1-6 cell lines. **(C)** The intracellular TG levels were quantitatively measured in Hep1-6 cells transfected with CD38 plasmid or vector under OA stimulation. **(D, E)** The lipid accumulation was measured by Oil red O staining in Hep1-6 cells treated with OA. **(F, G)** The expression of Sirt1 protein was determined by western blot analysis in Hep1-6 cells treated with OA. **(H-J)** The expressions of PPARα and CPT1 proteins were quantitatively determined by western blot analysis. **(K)** The expression of PPARα was examined by real-time PCR analysis in Hep1-6 cells treated with OA. Data are shown as means ± SEM, *p<0.05, **p<0.01 and ***p<0.001, n = 3 per group.

In order to further verify the roles of CD38 in hepatic lipid accumulation and oxidative stress, the stable Hep1-6 cell line overexpressing CD38 was obtained by transfecting CD38 expression plasmid and screening with G418 antibiotics. The overexpression of CD38 was confirmed by qPCR and Western blot (Figure 5A-5B) and the results showed that OA significantly increased the intracellular TG level, whereas overexpression of CD38 further enhanced OA-induced TG production compared with control (Figure 5C). The Oil Red O staining showed that overexpression of CD38 significantly increased OA-induced lipid accumulation (Figure 5D-E). In addition, the expression of Sirt1 was decreased in overexpressing CD38 cells after OA treatment (Figure 5F-G). Moreover, the expressions of the genes related to fatty acid oxidation such as PPARα and CPT1 were significantly decreased in Hep1-6 cells with overexpressing CD38 (Figure 5H-5K). These results indicated that overexpression of CD38 was able to aggravated lipid accumulation in hepatocytes *in vitro*.

### Overexpression of CD38 aggravates hepatocytic oxidative stress *in vitro*

**Figure 6:**
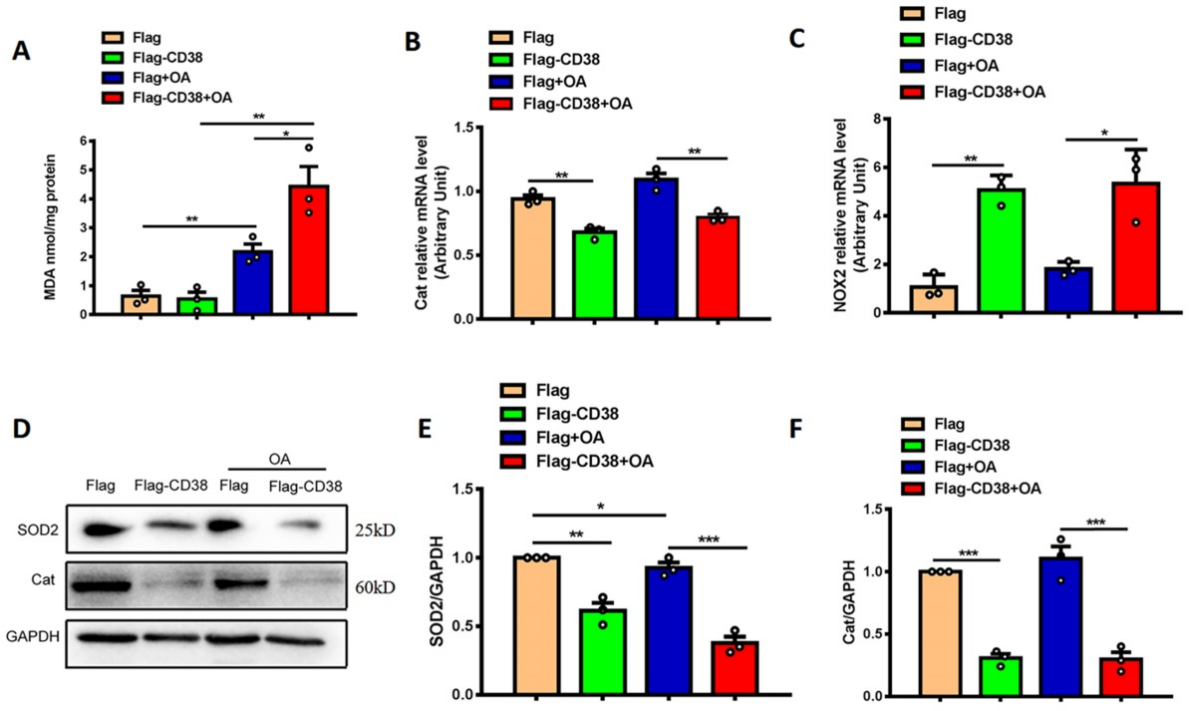
** Overexpression of CD38 aggravated OA-induced hepatocytic oxidative stress *in vitro*. (A)** The MDA formation was measured in Hep1-6 cells transfected with CD38 plasmid or vector under OA treatment. The mRNA expressions of Cat **(B)** and NOX2 **(C)** were measured by real-time PCR analysis in OA-induced Hep1-6 cells. The expressions of SOD2 **(D, E)** and Cat **(D, F)** proteins were determined by western blot analysis in Hep1-6 cells. Data are shown as means ± SEM, *p<0.05, **p<0.01 and ***p<0.001, n = 3 per group.

To further confirm whether CD38 affects OA-induced oxidative stress in hepatic cells *in vitro*, OA-induced oxidative stress was evaluated in Hep1-6 cells overexpressing CD38. The results showed that overexpression of CD38 significantly enhanced OA-induced MDA formations (Figure 6A), decreased the transcripts of Cat (Figure 6B) and increased the mRNA expression of NOX2 (Figure 6C) with or without OA stimulation. In addition, overexpression of CD38 remarkably inhibited the protein levels of SOD2 and Cat in Hep1-6 cells overexpressing CD38 with or without OA stimulation (Figure 6D-F). All these results indicated that overexpression of CD38 increased oxidative stress in hepatocytes *in vitro*.

### Sirt1 and Sirt3 specific inhibitors promote OA-induced lipid accumulation via suppressing fatty acid oxidation and enhancing oxidative stress in hepatocytes

**Figure 7:**
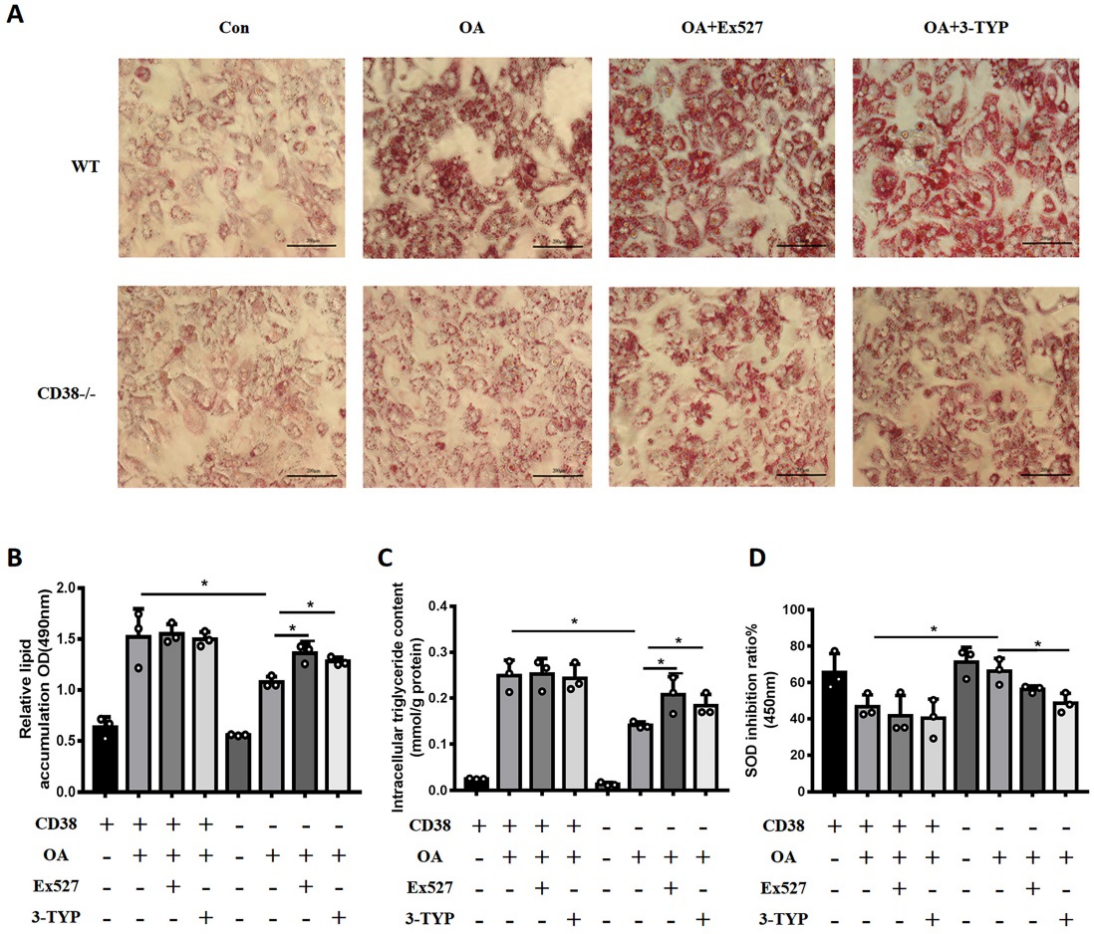
** Sirt1 and Sirt3 specific inhibitors promoted OA-induced lipid accumulation through suppressing fatty acid oxidation and oxidative stress in hepatocytes. (A)** The images of Oil red O staining in primary hepatocytes from WT and CD38^-/-^ mice treated with Ex527 or 3-TYP. Lipid formations were assessed by Oil Red O staining and visualized by bright field light microscopy in primary hepatocytes after treated with Ex527 (a Sirt1 inhibitor) and 3-TYP (a Sirt3 inhibitor). **(B)** Quantitative analysis of lipid formation. The relative lipid accumulation was quantitatively analyzed by measuring absorbance at 490 nm. **(C)** The intracellular TG levels were quantitatively measured in primary hepatocytes from WT and CD38^-/-^ mice treated with Ex527 or 3-TYP. **(D)** The SOD relative activity was determined in primary hepatocytes from WT and CD38^-/-^ mice treated with Ex527 and 3-TYP. Data are shown as means ± SEM, *p<0.05 and **p<0.01, n = 3 per group.

In order to clarify whether the anti-NAFLD of CD38 deficiency was related to the intracellular NAD^+^ decline, we examined the effects of Sirt1 and Sirt3 specific inhibitors on OA-induced lipid accumulation in primary hepatocytes from CD38^-/-^ mice. As showed in Figure 7A and 7B, both Ex527 (Sirt1 inhibitor) and 3-TYP (Sirt3 inhibitor) enhanced OA-induced lipid accumulation in primary hepatocytes from CD38^-/-^ mice although we did not observe that these inhibitors further promoted OA-induced TG accumulation in WT hepatocytes. More importantly, the results also showed that these inhibitors significantly promoted OA-induced the accumulation of TG and inhibited SOD activity (Figure 7C and 7D), suggesting that the anti-lipid accumulation of CD38 deficiency might be dependent on NAD-mediated enhancement of FAA β-oxidation and suppression of oxidative stress.

## Discussion

NAFLD is one of the most common liver diseases in the world. The earliest stage of NAFLD is steatosis which is characterized by accumulating a large amount of triglycerides in liver and it will be able to develop to hepatitis or cirrhosis if it is out of control 1. CD38 is a main hydrolase of intracellular NAD^+^ in mammalian cells, in which CD38 is involved in many biological processes. We previously demonstrated that CD38 deficiency protected against inflammation, obesity, cardiac hypertrophy, ischemia/reperfusion injury and cardiomyocyte senescence 11-14. More recently, we found that inhibition of NAMPT, a rate-limiting enzyme regulated NAD^+^ biosynthesis, aggravated HFD-induced hepatic steatosis 19. However, whether deletion of CD38 protects mice from HFD-induced NAFLD and the underlying mechanism remains unknown. In the present study, we observed that HFD or OA significantly increased the expression of CD38 in hepatic tissues or hepatocytes *in vivo* and *in vitro*, respectively, suggesting that CD38 may play an important role in NAFLD. It has been reported that the expression of CD38 was regulated by many transcriptional factors such as nuclear receptor superfamily, signal transducer and activator of transcription (STAT), nuclear factor (NF-κB), activator protein 1 (AP-1), and inflammatory mediators or cytokines such as LPS and TNFα 20-22. In addition, some microRNAs such as miR-140-3p also regulated the TNF-α-induced CD38 expression 23 at post-transcriptional level. In the present study, we observed that HFD was able to upregulate the expression of CD38 in hepatic tissues, suggesting that CD38 might play a role in HFD-induced NAFLD. However, the mechanisms of HFD-induced the expression of CD38 was not explored, which can be further investigated in the future. As we expected, deletion of CD38 significantly alleviated HFD-induced hepatic steatosis via reducing lipid accumulation and oxidative stress in liver.

PPARα is a key factor for regulating fatty acid oxidation, and PPARα deficiency aggravated multiple factors-induced hepatic steatosis in the development of NAFLD through attenuating fatty acid oxidation 24-27. In the present study, we found that CD38 deficiency alleviated HFD or OA-induced lipid accumulation in liver tissues or hepatocytes by increasing the expression of PPARα. Fatty acid oxidation in liver mainly occurs in the mitochondria, and its transport is regulated by the carnitine translocase CPT1 and CPT2. In the present study, we observed that the expressions of β-oxidation related genes such as CPT1 and ACOX1 were increased in CD38^-/-^ mice, suggesting that CD38 deficiency attenuated fatty acid oxidation through increasing expressions of CPT1 and ACOX1. Furthermore, the anti-hepatic steatosis of CD38 deficiency was further confirmed with primary hepatocytes and Hep1-6 cells overexpressing CD38 *in vitro*. Taken together, our results demonstrated that CD38 deficiency protected hepatocytes from HFD- or OA-induced lipid accumulation through modulating fatty acid oxidation *in vivo* and *in vitro*.

“Two hits hypothesis” is used to clarify the development of NAFLD. The “first hit” is due to the excessive accumulation of TG in liver tissues, and the “second hit” makes the simple steatosis further develops into NASH, such as oxidative stress 28. Oxidative stress is associated with lipid peroxidation, inflammation, cytokine activation and the production of reactive oxygen species 29. Intracellular GSH/GSSH is an effective indicator for evaluating oxidative stress. We found that CD38 deficiency significantly reduced the ratio of GSH/GSSG in liver tissues from HFD treated mice, suggesting that lack of CD38 was able to reduce oxidative stress. Malondialdehyde (MDA) is an end-product of lipid peroxidation, changes in the content of MDA can be used to assess oxidative stress damage 30. In the present study, we found that CD38 deficiency significantly reduced OA-induced increases of MDA in liver tissues or hepatocytes. In contrast, overexpression of CD38 increased the content of MDA in hepatocytes. Superoxide dismutase (SOD) is one of the important enzyme for scavenging free radical species 31. In the current study, we observed that CD38 deficiency increased antioxidant gene SOD2 expression, while over-expression of CD38 in Hep1-6 cells increased the expression of NOX2 and decreased the expression of Catalase. These results indicated that the increase of the GSH/GSSG ratio in CD38 deficiency livers was one of the mechanisms for decreasing oxidative stress induced by HFD. At the same time, CD38 deficiency reduced oxidative stress damage by decreasing MDA formation and increasing SOD activity *in vivo* and *in vitro*, preventing the further development of NAFLD.

Sirtuins proteins are a family of NAD^+^-dependent deacetylases that participate in the regulations of many biological processes including liver glucose and fatty acid metabolism, mitochondrial energy production, liver gluconeogenesis, insulin secretion and fat cell maturation 32-35. It has been reported that deletion of Sirt1 in hepatocytes resulted in hepatic steatosis and inflammation 26, indicating that Sirt1 played an important role in NAFLD. On the contrary, overexpression of Sirt1 protected mice from hepatic steatosis and liver inflammation induced by HFD 36. These results demonstrated that Sirt1 played an important role in NAFLD. In our study, we observed the expressions of Sirt1 and its target genes such PPARα and SOD2 were upregulated in CD38 deficiency mice and hepatocytes treated with HFD and OA. More importantly, Ex527 (Sirt1 inhibitor) and 3-TYP (Sirt3 inhibitor) significantly aggravated OA-induced the increase of TG and inhibited SOD activity, suggesting that the anti-lipid accumulation of CD38 deficiency is dependent on NAD-mediated enhancement of FAA β-oxidation and suppression of oxidative stress. Taken together, our results demonstrated that CD38 deficiency protected mice from HFD-induced NAFLD through activating Sirtuins/PPARα/SOD2 signaling pathway.

In conclusion, the results from our present study demonstrated that CD38 deficiency prevented HFD-induced NAFLD, and the underlying mechanisms were mainly related to activating Sirtuins/PPARα/SOD2 signaling pathway. Obviously, our findings provided an insight in elucidating the underlying mechanisms of NAFLD development and the CD38 may be a possible target for preventing liver steatosis and NAFLD.
